# Use of human intra-tissue stem/progenitor cells and induced pluripotent stem cells for hair follicle regeneration

**DOI:** 10.1186/s41232-019-0093-1

**Published:** 2019-02-25

**Authors:** Manabu Ohyama

**Affiliations:** Department of Dermatology, Kyorin University Faculty of Medicine, 6-20-2 Shinkawa, Mitaka, Tokyo, 181-8611 Japan

**Keywords:** Hair follicle, Regeneration, Epithelial-mesenchymal interactions, Stem cell, Progenitor cell, Induced pluripotent stem cell

## Abstract

**Background:**

The hair follicle (HF) is a unique miniorgan, which self-renews for a lifetime. Stem cell populations of multiple lineages reside within human HF and enable its regeneration. In addition to resident HF stem/progenitor cells (HFSPCs), the cells with similar biological properties can be induced from human-induced pluripotent stem cells (hiPSCs). As approaches to regenerate HF by combining HF-derived cells have been established in rodents and a huge demand exists to treat hair loss diseases, attempts have been made to bioengineer human HF using HFSPCs or hiPSCs.

**Main body of the abstract:**

The aim of this review is to comprehensively summarize the strategies to regenerate human HF using HFSPCs or hiPSCs. HF morphogenesis and regeneration are enabled by well-orchestrated epithelial-mesenchymal interactions (EMIs). In rodents, various combinations of keratinocytes with mesenchymal (dermal) cells with trichogenic capacity, which were transplanted into in vivo environment, have successfully generated HF structures. The regeneration efficiency was higher, when epithelial or dermal HFSPCs were adopted. The success in HF formation most likely depended on high receptivity to trichogenic dermal signals and/or potent hair inductive capacity of HFSPCs. In theory, the use of epithelial HFSPCs in the bulge area and dermal papilla cells, their precursor cells in the dermal sheath, or trichogenic neonatal dermal cells should elicit intense EMI sufficient for HF formation. However, technical hurdles, represented by the limitation in starting materials and the loss of intrinsic properties during in vitro expansion, hamper the stable reconstitution of human HFs with this approach. Several strategies, including the amelioration of culture condition or compartmentalization of cells to strengthen EMI, can be conceived to overcome this obstacle. Obviously, use of hiPSCs can resolve the shortage of the materials once reliable protocols to induce wanted HFSPC subsets have been developed, which is in progress. Taking advantage of their pluripotency, hiPSCs may facilitate previously unthinkable approaches to regenerate human HFs, for instance, via bioengineering of 3D integumentary organ system, which can also be applied for the treatment of other diseases.

**Short conclusion:**

Further development of methodologies to reproduce *bona fide* EMI in HF formation is indispensable. However, human HFSPCs and hiPSCs hold promise as materials for human HF regeneration.

## Background

**Fig. 1:**
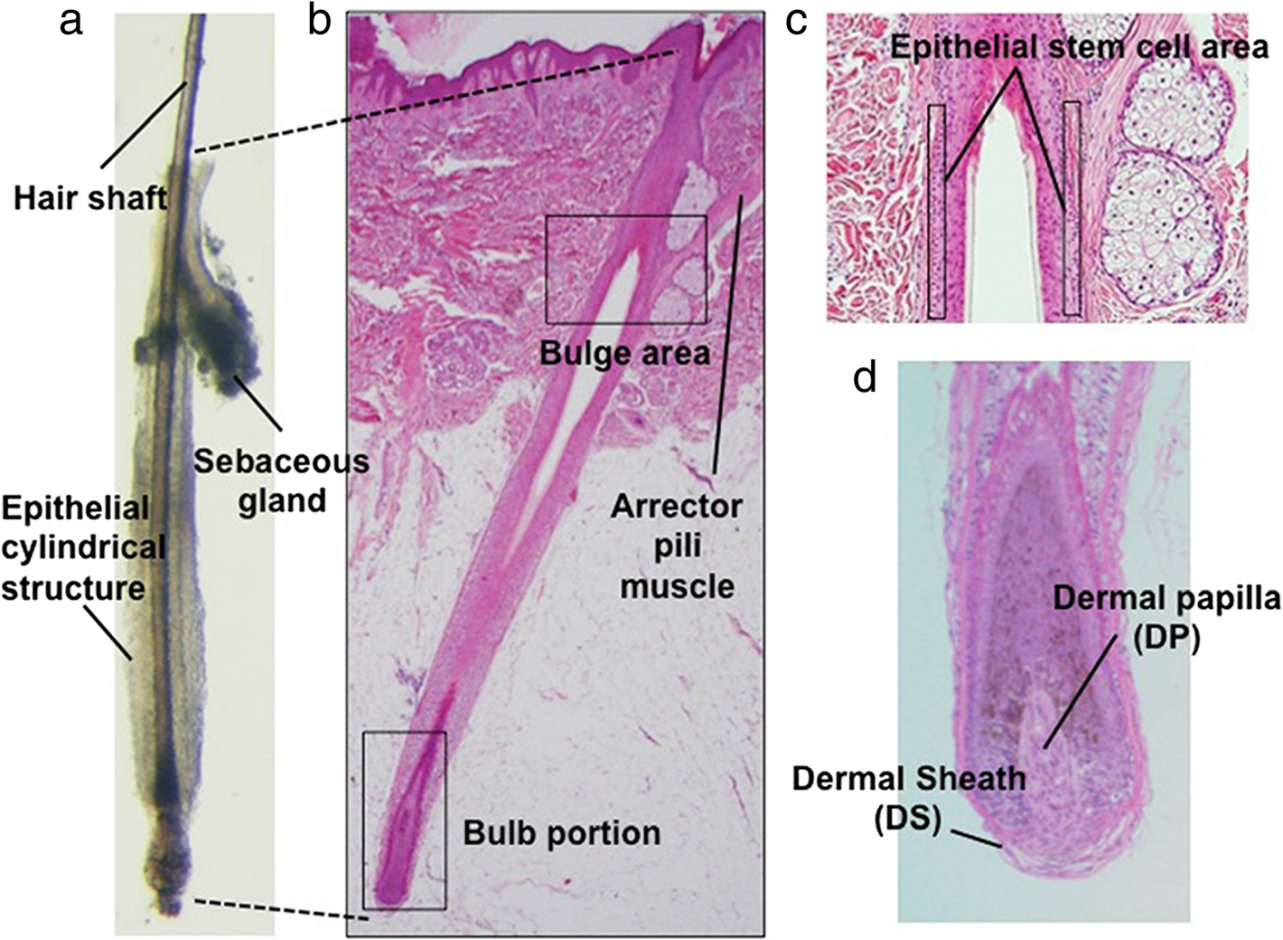
Normal human scalp hair follicle structure. **a** A hair follicle microdissected from human scalp (an anagen hair follicle is presented). **b** Corresponding histopathology image. The bulge area harbors stem cells. Hair matrix cell proliferation in the bulb results in hair shaft elongation. **c** Hair follicle epithelial stem cells locate in the outermost layer of the outer root sheath. **d** The dermal papilla (DP) and the dermal sheath (DS) are mesenchymal components demonstrate trichogenic activity

The hair follicle (HF) is a skin appendage that mainly consists of cylindrical multiple layers of keratinocytes surrounding the hair shaft with a specialized mesenchymal cell aggregate of the dermal papilla (DP) at its proximal end (Fig. 1a, b) [1]. In humans, HF not only provides physical and immunological barrier for external insults [1–3] but also impacts on one’s appearance. Thus, a huge demand for the treatment of hair loss conditions exists and numerous approaches with varying levels of evidence have been developed. With recent advances in regenerative medicine, especially the emergence of human induced pluripotent stem cells (hiPSCs), the possibility of regenerating human HF has been globally discussed [4].

In fact, human HF regeneration for treating non-autoimmune-mediated hair loss diseases, such as androgenetic alopecia or female pattern hair loss, may serve as an ideal model to probe the feasibility of regenerative medicine approaches for several reasons: (1) HF is easily accessible and observable; (2) HF morphogenesis, biology, and physiology have been well understood; (3) in vitro maintenance and cultivation of HF or HF-derived cells have been established; (4) at least in rodents, the methodologies to reconstitute HFs in vivo have been established; and (5) autologous transplantation of HFs in bald area has been widely conducted, etc. [3–6]. Of note, HF is a periodically self-renewing miniorgan harboring multiple stem/progenitor cell populations represented by epithelial HF stem cells (HFSCs) at the bulge area (Fig. 1c) and DP or its precursors in the dermal sheath (DS) (Fig. 1d), which serve as ideal cell sources for HF regeneration and, potentially for hiPSC generation [4–6]. Ultimately, HF-derived hiPSCs can be converted into HFSCs and unlimitedly supply materials for human HF regeneration [7].

**Fig. 2:**
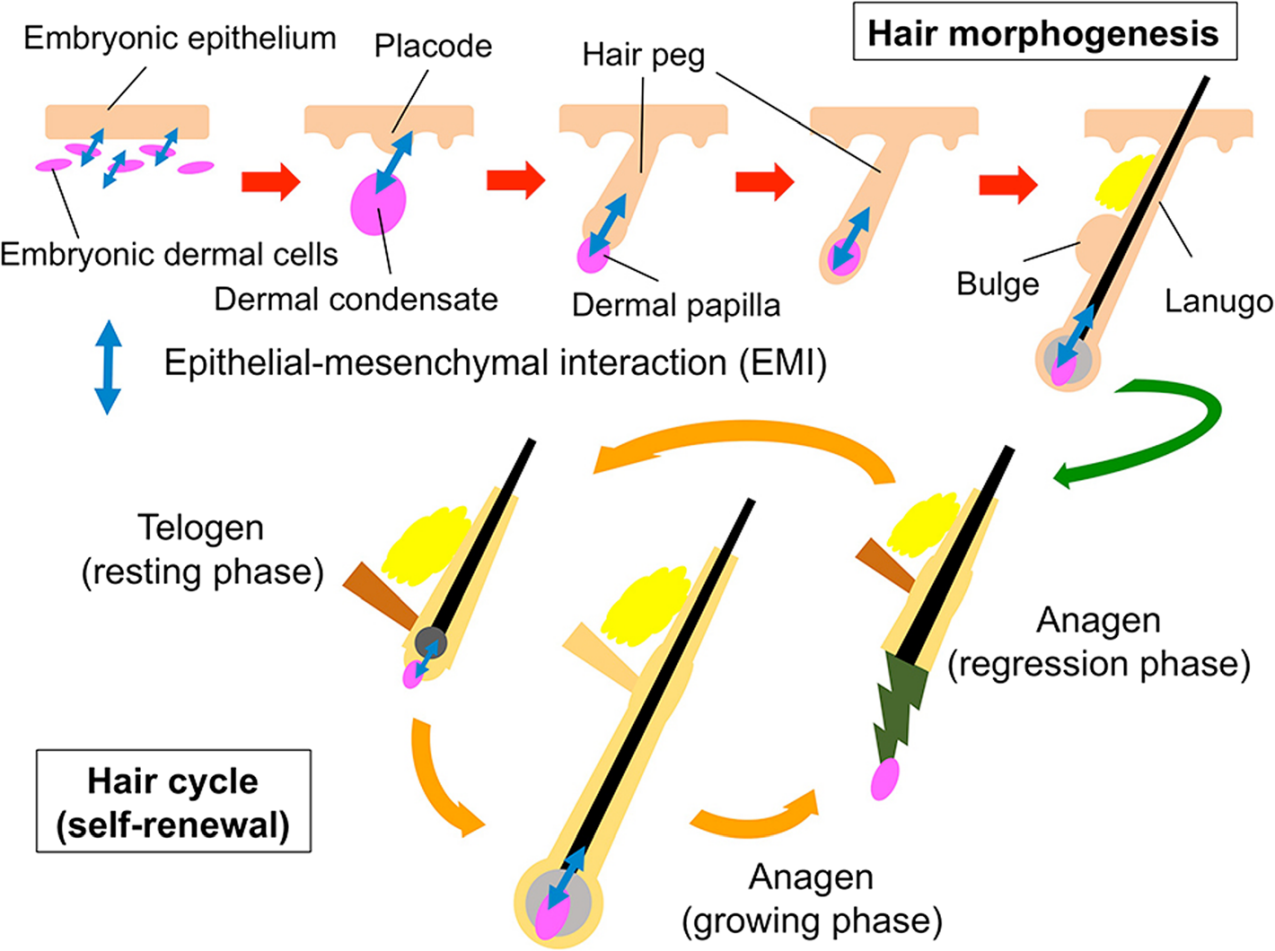
Hair follicle morphogenesis and hair cycle Hair follicle morphogenesis and hair cycle are enabled by well-orchestrated epithelial-mesenchymal interactions. In morphogenesis, crosstalk between the placode and the dermal condensate (the precursor of the dermal papilla) initiates epithelial invagination to form the lanugo. In hair cycle, communication between bulge stem cells and the dermal papilla is thought to play key roles in telogen (resting phase)-anagen (growing phase) transition

HF morphogenesis and regeneration depends on intensive and well-orchestrated interactions between receptive epithelial and inductive mesenchymal components (Fig. 2) [3, 5, 8]. In the past attempts to bioengineer HF, variously prepared epithelial and mesenchymal components were combined and grafted into a permissive in vivo environment to elicit intercompartmental interactions [5, 6]. Theoretically, less-committed and highly proliferative HFSCs or progenitor cells could efficiently yield HFs in those conventional assays. In line with this hypothesis, HFSCs were shown to be favorable materials for HF regeneration, at least, in rodents [9, 10]. However, the use of human HFSCs or progenitor cells for such application was hampered by the limitation in collectable cells and the loss of their intrinsic properties during in vitro expansion [4]. Improvement of culture condition to maintain/restore their intrinsic property is pivotal.

Recent studies support the usefulness of hiPSCs to experimentally regenerate human HF [11–14]. The cell populations biologically resembling epithelial HFSCs or DP cells can be induced from hiPSCs [12, 14, 15]. Co-grafting with either component respectively with dermal or epithelial cells into in vivo environment resulted in HF-like structure formation. Furthermore, hiPSCs may enable previously unthinkable approaches to bioengineer HFs. For instance, taking advantage of their pluripotency, 3D integumentary organ system can be directly generated from hiPSCs [13].

The aim of this review is to summarize the strategies for using human HFSCs and progenitor cells or hiPSCs for HF regeneration with a particular emphasis on the enhancement of epithelial-mesenchymal interactions (EMIs).

## Experimental techniques to elicit folliculogenic EMI

Various approaches have been attempted to elicit EMIs sufficient for HF regeneration [4]. However, all assays are based on the same principle; combining responder epithelial cells with inducer mesenchymal cells that are placed into a neutral permissive environment [5]. A two-step approach consisting of in vitro experimentations to establish a condition to maximize EMIs and in vivo HF reconstitution assays adopting materials prepared in the condition optimized in vitro study would be beneficial [4, 5].

### In vitro approaches

Organ culture of microdissected human HFs could be one of best approaches to monitor in vivo EMIs [16]. However, HF isolation from the scalp sample can be laborious and the specimen is not readily available for all institutions.

**Fig. 3:**
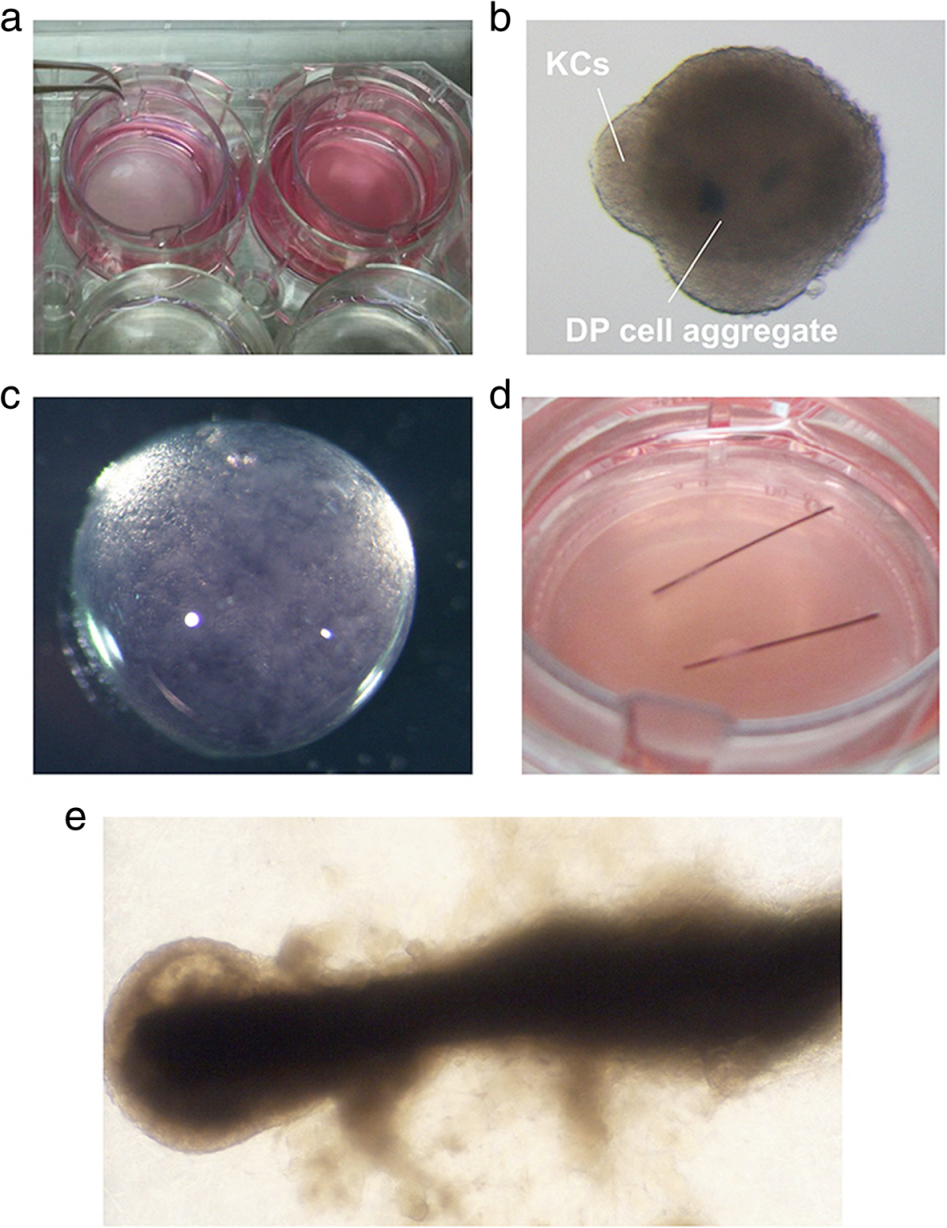
In vitro approaches to elicit folliculogenic EMI. **a** Co-culture of hair follicle epithelial and mesenchymal components using cell inserts. **b** Formation of spheres in which keratinocytes (KCs) are covering a dermal papilla (DP) cell aggregate. **c** Compartmentalization of KCs and DP cells in a collagen gel droplet. **d** Reproduction of hair follicle-like structures made of human KCs and DP cells in Matrigel. **e** Regenerated HF-like structure in Matrigel. The data shown in (**d**, **e**) are obtained in a preliminary experiment supported by JSPS KAKENHI (Grant Number JP 16H05370 to MO)

More simply, HF keratinocytes (HFKCs) and DP, DS, or other trichogenic dermal cells (e.g., murine neonatal fibroblasts) can be co-cultured using cell inserts (Fig. 3a) [4, 5]. Epithelial and mesenchymal components crosstalk via shared culture medium with resultant HFKC proliferation, HFKC-related gene (e.g., *KRT15, CD200, DIO2, GATA3, TRPS1, KRT75, MSX2*) upregulation, or DP biomarker (e.g., *ALPL, VCAN, LEF1,* *WNT5*,* NOG, SPRY4*) expression within a couple of days [11, 17, 18]. Major drawback of this approach is the absence of direct intercompartmental crosstalk enabled by cell-cell contact.

To overcome this drawback, KCs and DP or equivalent cells can be mixed to form cell aggregates and maintained in 3D culture condition (Fig. 3b) [19–21]. Magnitude of EMIs can be measured by the expression levels of HFKC or and DP marker expression [19–21]. EMI can further be enhanced by cell compartmentalization methods, in which HF epithelial and mesenchymal cells were placed at high-density in acid-soluble collagen allowing the cells to elicit sufficient EMIs (Fig. 3c) [22]. Taking advantage of this idea, cylindrically assembled human KCs can be placed onto human DP cell aggregates embedded within collagen gel, allowing formation of a structure partially reproducing HF microanatomy in vitro in pilot studies (Fig. 3d, e).

In hair morphogenesis, focal thickening of the embryonic epidermis, i.e., hair placode, and DP precursors in the dermis communicate to initiate HF formation (Fig. 2). To mimic this situation in vitro, we are currently attempting to establish an assay in which trichogenic dermal cell or tested cell aggregates are embedded into the epidermal-dermal interface of three-dimensional human skin culture. A preliminary data suggests that the detection of some HF morphogenesis-related genes is possible under this experimental condition.

### In vivo approaches

**Fig. 4:**
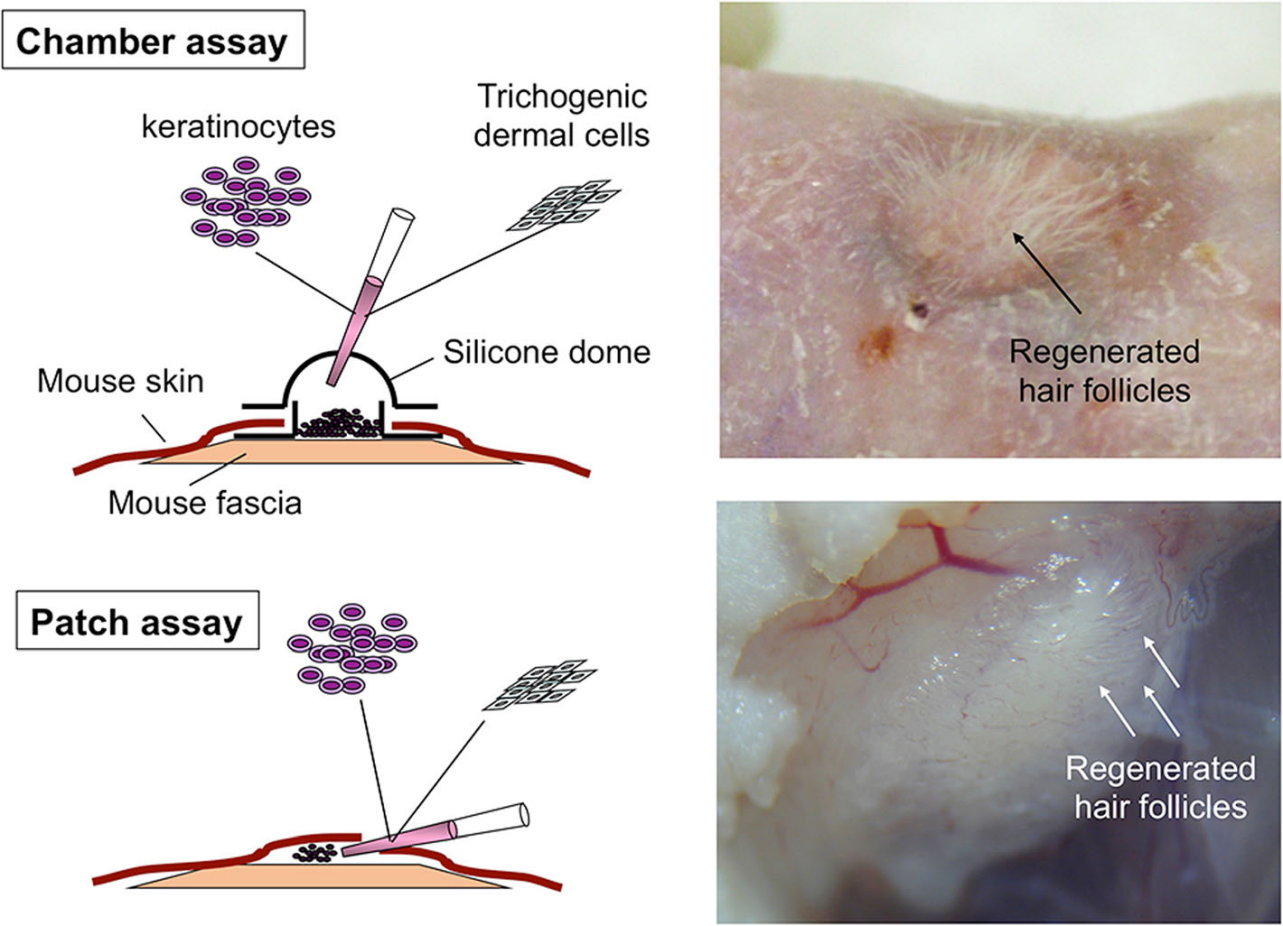
Approaches for HF regeneration in vivo. In the chamber assay, mixture of keratinocytes and trichogenic dermal cells are transplanted into a silicone chamber on the back of immunodeficient mice. In the patch assay, cell mixture is directly injected into the hypodermis of immunodeficient mice

Considering that HF regeneration efficiency should correlate well with the intensity of EMI, in vivo HF reconstitution assays adopting cell transplantation into immunodeficient mice would be the most optimal way to trigger EMI (Fig. 4) [5]. In the chamber assay, mixture of KCs and trichogenic dermal cells (mostly DP, DS, or neonatal dermal fibroblasts) were transplanted into a silicon chamber grafted on to the dorsal fascial surface (Fig. 4). The chamber keeps the humidity and space enabling HF reconstitution. Newly generated HFs can be visible from the outside of the body. However, larger number of the cells and longer period is necessary for this assay, when compared to the path assay, in which epithelial and dermal cell mixture is directly injected into the hypodermis of mice (Fig. 4). The former requires 10 million or more cells and around 35 days to see HFs, while the latter requires around 1 million cells and around 10 days (summarized in [5]). In addition, a large regenerated HF-bearing area can be observed in the chamber assay, but several patches can be generated in a single mouse in the patch assay (Fig. 4). Because of the limitation in human samples usable for experimentations and the technical simplicity, the patch technique may be favorable for attempts to regenerate HF using human-derived cells [11]. The sandwich assay is also used to evaluate hair inductive capacity of dermal cells [5, 18]. In this assay, tested dermal cell aggregates were implanted between the epidermis and dermis of globular skin piece, which is subsequently grafted in the subcutaneous space. When HF or similar structure is formed, the transplanted dermal cells can be considered to possess trichogenic activity [5, 18]. In addition to those assays, some derivatives with minor modifications have been reported with varying levels of success (summarized in [5]).

## Use of epithelial stem/progenitor cells to increase HF formation efficiency

**Fig. 5:**
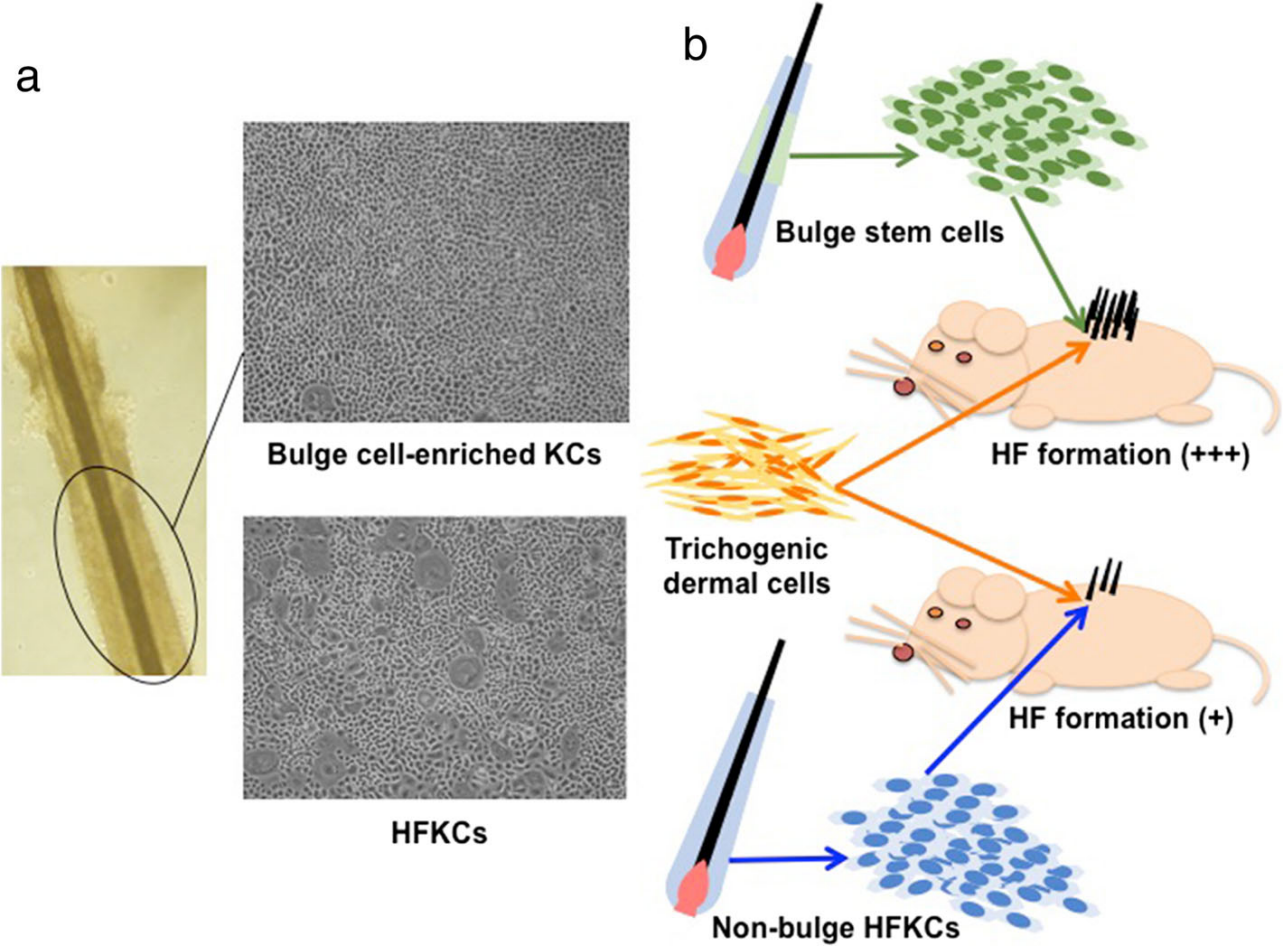
Use of epithelial stem/progenitor cells to increase HF formation efficiency. **a** Isolated human HF bulge cell-enriched keratinocytes (KCs) are highly proliferative when compared to non-bulge HFKCs. **b** The comparison of hair forming capacity between bulge stem cells (SCs) and non-bulge HFKCs. The same number of bulge SCs and non-bulge HFKCs were isolated form HFs and co-transplanted with the same amount of trichogenic dermal cells (usually mouse neonatal dermal fibroblasts) into immunodeficient mice to assess HF forming efficiency

Previous studies adopting in vivo assays as described above indicated that the efficiency of HF regeneration and the morphology of regenerated structures were greatly influenced by the type of epithelial cell components under the condition that the identical trichogenic dermal cells were used as hair induction drivers [10, 23–25]. Past studies demonstrated that multiple epithelial cell subsets with high proliferative capacity and multipotency to regenerate multiple lineage of the pilosebaceous unit exist within HF [9, 10, 26–30]. Keratin 15 high-expressing slow-cycling HF epithelial stem cells residing in the bulge area of the outer root sheath, an insertion point of the arrector pili muscle, is the most established “HFSC” subset, which potentially provide optimal materials for HF regeneration (Fig. 5a, b) [31–33]. In line with this, isolated keratin 15 and CD34-positive murine bulge cells more efficiently reconstituted complete HF structures than non-bulge HFKCs, when co-grafted with identical dermal cells into in vivo environment [9, 10]. This finding supported the hypothesis that epithelial HFSCs are more receptive for dermal trichogenic signal and represent better material for HF bioengineering [4].

Whether or not this observation is applicable for human subjects has not been fully addressed. Although CD200 was identified as a cell surface marker, which enables the isolation of highly proliferative human bulge cells [33], the bulge cells obtainable from clinical samples are usually limited and insufficient to conduct even the patch assay. In addition, human epithelial HFSCs seemed to lose their intrinsic properties when they were cultured as demonstrated by the downregulation of signature genes, including keratin 15 [32], CD200 [33], *Lhx2* [34], and *Sox9* [35]. How this affects their ability to communicate with mesenchymal cells needs to be appropriately investigated. However, unlike murine epithelial HFSCs, use of human counterpart to regenerate HFs is still technically challenging.

A possible approach to overcome this issue would be to increase the receptivity of KCs to trichogenic dermal signals by predisposing them to follicular fate. Activation of Wnt/β-catenin pathway may be a promising approach [36–38] as forced expression of β-catenin in the epidermis resulted in ectopic expression of hair keratins or de novo hair follicle formation in mice [39, 40]. Modulation of p63 expression in KCs may also enhance the response to trichogenic dermal message to the level analogous to that in HFSCs [41]. Yet, an extreme caution needs to be paid for adopting these strategies for human HF regeneration, as aberrant expression of such genes may result in tumor formation. For instance, overactivation of β-catenin could give rise to pilomatricoma [42].

Amelioration of culture condition to maintain HFSC properties would be useful to prepare large number of HFSCs for HF bioengineering. A recent study demonstrated that murine HFSCs could be expanded maintaining their biological characteristics including high HF forming capacity when they were cultured three-dimensionally in Matrigel containing ROCK inhibitor (Y27632), FGF-2, and VEGF-A [43]. How this methodology sustains human HFSC properties in vitro is still unclear and needs to be investigated in future studies.

An alternative approach to enhance KC receptivity to dermal signal is to use neonatal or embryonic KCs. Past in vivo grafting studies demonstrated that neonatal or fetal KCs were able to regenerate HF or HF-like structures [24, 44, 45]. Some HF-forming capacity could still be observed after cultivation of fetal cells. Apparently, this strategy cannot be directory adopted for clinical applications; however, these observations can drop a hint for enhancing EMIs for HF regeneration. Human adult KCs can reacquire some juvenile properties by basic fibroblast growth factors treatment [46]. Likewise, exposure of KC to major factors playing key roles in the early phase of HF morphogenesis may allow KCs to exhibit HF forming cell (e.g., hair placode cell) phenotype. WNT, Ectodysplasin-A (EDA), BMP, and sonic hedgehog (SHH) signaling pathways are involved in HF placode formation [3, 8]. Either activation or suppression of these pathways in cultured KCs by supplementation of ligands could endow the cells with some HFSC properties. Feasibility of this approach is under investigation using human 3D skin equivalents and preliminary data suggested upregulation of several hair placode signature genes could be achieved.

## Preparation of trichogenic dermal cells for successful HF induction

**Fig. 6:**
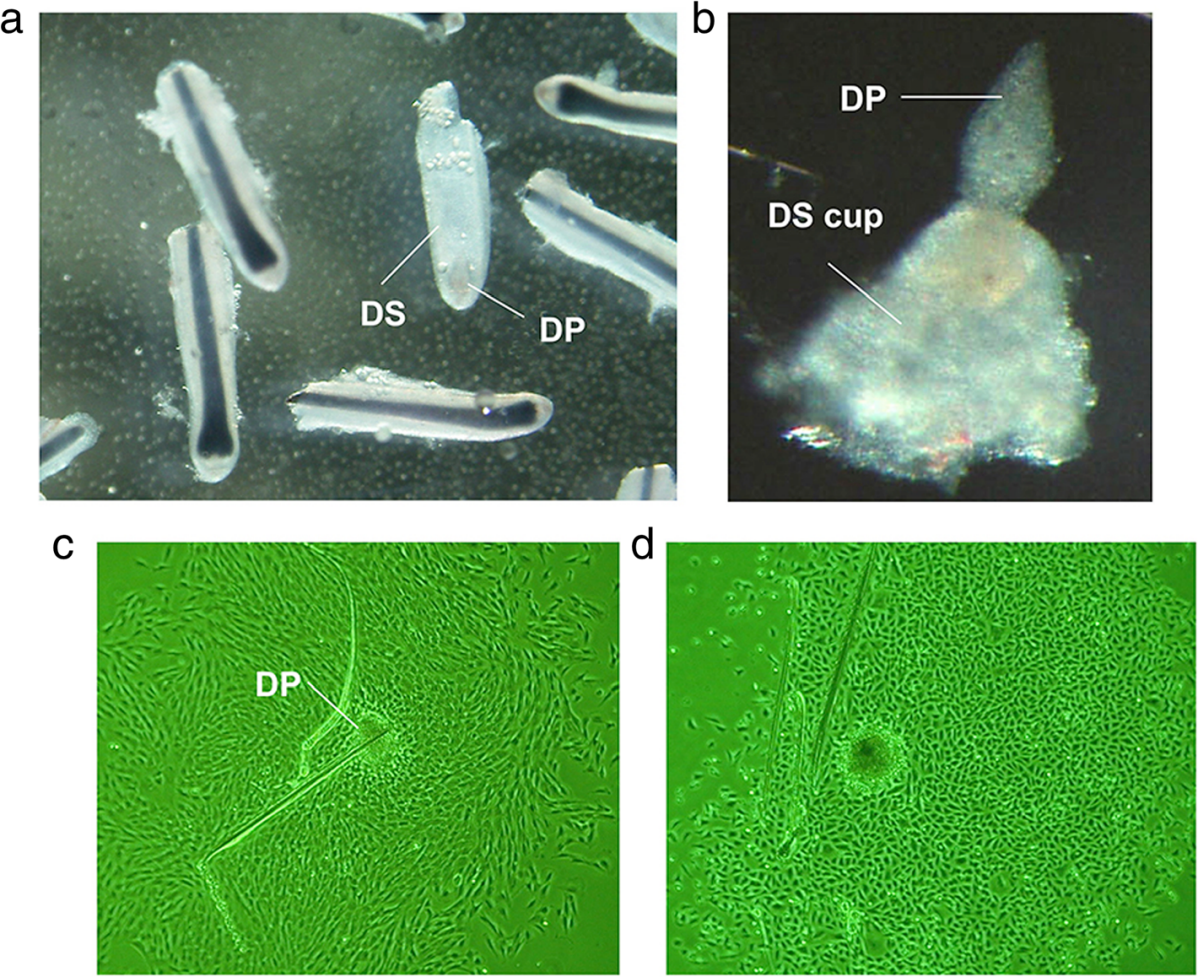
Preparation of trichogenic dermal cells for successful HF induction. **a** Microdissected lower portion of human HFs. The dermal papilla (DP) and the dermal sheath (DS) are respectively indicated. **b** High magnification image of DP and the “flipped” cup shape portion of DS. Note that two components are continuous. **c** In vitro expansion of human DP. **d** Restoration of DP properties in DP activation culture condition (DPAC). Note that DP cell morphology is distinct from that shown in (**c**).

In HF, DP cells or DS cells locating closely to DP in the cup-shaped HF end are shown to possess hair inductive capacity (Fig. 6a, b) [5]. In pioneering studies, surgical removal of DPs from vibrissa HFs resulted in the arrest of hair shaft elongation [47], while transplantation of microdissected DPs or DS cells into recipient skin successfully induced HFs [48], clearly indicating the indispensable role of those cells in HF morphogenesis and regeneration. Therefore, preparation of sufficient amount of DP, DS, or dermal cells with equivalent hair inductive capacity is essential to achieve successful HF bioengineering.

### Strategies to prepare DP cells while enhancing hair inductive capacity

DP cells are the most representative trichogenic dermal cells [5]. Murine DP cells which can be efficiently isolated by cell sorter using genetically introduced fluorescent protein or CD133 as the cell surface marker [49–51], while human DP cells are usually manually microdissected from scalp samples (Fig. 6b) and expanded in vitro (Fig. 6c) before further experimentation as a specific surface marker is not readily available [5]. The major problem of DP cell culture is, similar to that of KC culture, the loss of intrinsic properties during in vitro expansion [18, 52, 53] and various approaches have been attempted to overcome this hurdle.

In the bulb portion of HF, DP cells are maintained in a milieu of secreted ligands, growth factors, hormones, and extracellular matrices, which enables DP cells to crosstalk with HF matrix KCs and other cell subsets to sustain their properties [5]. Significance of this EMI has been supported by the observation that the exposure to KC-conditioned medium facilitates DP cells to sustain their intrinsic properties, such as signature gene expression and, more importantly, hair inductive capacity [54, 55]. Thus, supplementation of DP cell activating factors missing in conventional culture medium could sustain/restore DP properties in vitro. In line with this speculation, agonist-driven activation of key signaling pathways, which were downregulated in DP cells during in vitro expansion, such as WNT, BMP, TGF-β2, and FGF signaling pathways, resulted in amelioration of murine and human DP properties [18, 53, 56–58]. The effect can be synergistically enhanced by combination of individual factors. For instance, dermal papilla activation culture condition (DPAC) containing WNT, BMP, and FGF agonists has been established to sustain or elicit human DP cell properties in culture (Fig. 6d). Transplantation of DPAC-treated human DP cells and human KCs into nude mice resulted in incomplete but distinguishable HF-like structure formation [18].

Aggregative behavior represents a major biological feature of DP cells [18, 59–62] (Fig. 4c). Most likely because cell aggregation restores cell-cell contact enabling better crosstalk between cells, three-dimensional culture can “reprograms” microenvironment of DP cells and ameliorate biological characteristics of cultured DP cells [18, 63]. Indeed, DP cell aggregates expressed higher levels of DP biomarkers and exhibit greater in vivo hair inductive capacity than non-aggregated cells [18, 59–61]. Several methodologies, such as hanging drop culture [61], forced cell aggregation by centrifuging in a low-cell binding plate [18, 59], and self-assembly on poly (ethylene-co-vinyl alcohol) (EVAL) membranes [60] have been reported to be effective in generating DP spheres with respective biological effects.

The observations described above suggested the possibility of overcoming technical obstacles which hamper the preparation of sufficient trichogenic human DP cells to trigger intense EMI by modulating culture condition [4]. At the same time, currently available approaches would not allow preparation of fully competent human DP cells in vitro [18]. Further improvements, including activation of SHH signaling pathway [64] or addition of DP-associated extracellular matrices (e.g., laminin, type IV collagen, fibronectin, olfactomedin, versican [5]), may be beneficial to achieve full reactivation of cultured human DP cells for HF bioengineering. Overexpression of transcription factors associated with HF neogenesis including Sox2 or Tbx18 [65, 66] in functionally impaired DP cells may provide strategy to restore mouse DP cells; however, such approach would not be suitable for clinical appreciations because of potential risk of tumorigenesis.

### Preparation of DS cells as potential substitute for DP cells

Hair inductive capacity of DS cells, especially those in the proximal end of HF, has been demonstrated by various studies (summarized in [5]). DS tissue transplantation to amputated vibirissa HF restarted hair shaft production and implantation of human DS cells into the forearm skin of human recipient resulted in HF neogenesis [48, 67]. The classic observation that regeneration of DP in dissected upper two-third of vibrissa HFs [68] suggested that DS might contain DP stem cells or precursors, which was experimentally shown by in vivo fate mapping of DS cells [69]. Thus, DS represents an alternative candidate to elicit EMI for HF regeneration. In fact, currently ongoing clinical study adopts DS “cup” cells adjacent to DP obtained from microdissected human HF for the treatment of androgenetic alopecia (https://replicel.com/patients/hair-study).

### Elicitation of trichogenic activity in other mesenchymal cells

Neonatal mouse dermal fibroblasts have been routinely used in vivo HF reconstitution assay and are widely used for hair reconstitution assays [52, 70], implying that trichogenic capacity may be induced in mesenchymal cells other than DP or DS cells. Skin-derived precursor cells (SKPs) isolated from the dermis express Sox2 and nestin and shown to be trichogenic in mouse [71]; however, partly because isolation would be laborious, to what extent SKPs contribute to treatment of hair loss by HF regeneration is unclear. Adipose-derived stem cells (ASCs) could provide favorable cell source for various clinical applications. Rat ASCs can be combined with DP cells to form DP-like spheres, which exhibited superior DP characteristics than DP cell spheres [72]. Introduction of platelet-derived growth factor-A, SOX2, and beta-catenin genes can endow ASCs hair inductive capacity [73]. Instead of such genetic modifications, treatment of ASCs with a cocktail of apolipoprotein-A1, galectin-1, and lumican, the extracellular proteins overrepresented in embryonic perifolliculogenetic dermis [74], may enhance DP cell properties in ASCs.

## Approaches for HF regeneration using of human-induced pluripotent stem cells

Human-induced pluripotent stem cells (hiPSCs) hold great promise as material for regenerative medicine [7]. Intriguingly, cellular components of HF, such as HFKCs, melanocytes, and DP cells, can be reprogrammed into hiPSCs (summarized in [7]). HFKCs efficiently give rise to hiPSCs, which can be further differentiated into other cell types to form functional structures, for instance, neural progenitors and neurons [75]. DP cells and melanocytes express high levels of *SOX2* [76, 77]. Murine DP cells also upregulate *Klf4* and, taking advantage of intrinsic high expression of tow Yamanaka factors, can be differentiated into iPSCs with *Oct4* alone [77]. With current technology, four Yamanaka factors were indispensable to reprogram human DP cells into hiPSCs [63]; however, these findings support the idea of bioengineering large number of human HFs from a couple of HFs plucked from the scalp via generating hiPSCs and program those cells into HFKCs and DP cells. To date, several studies have reported the use of hiPSCs for the attempts to regenerate HF structures [11, 12, 14, 15].

### Generation of HF epithelial cells from hiPSCs

**Fig. 7:**
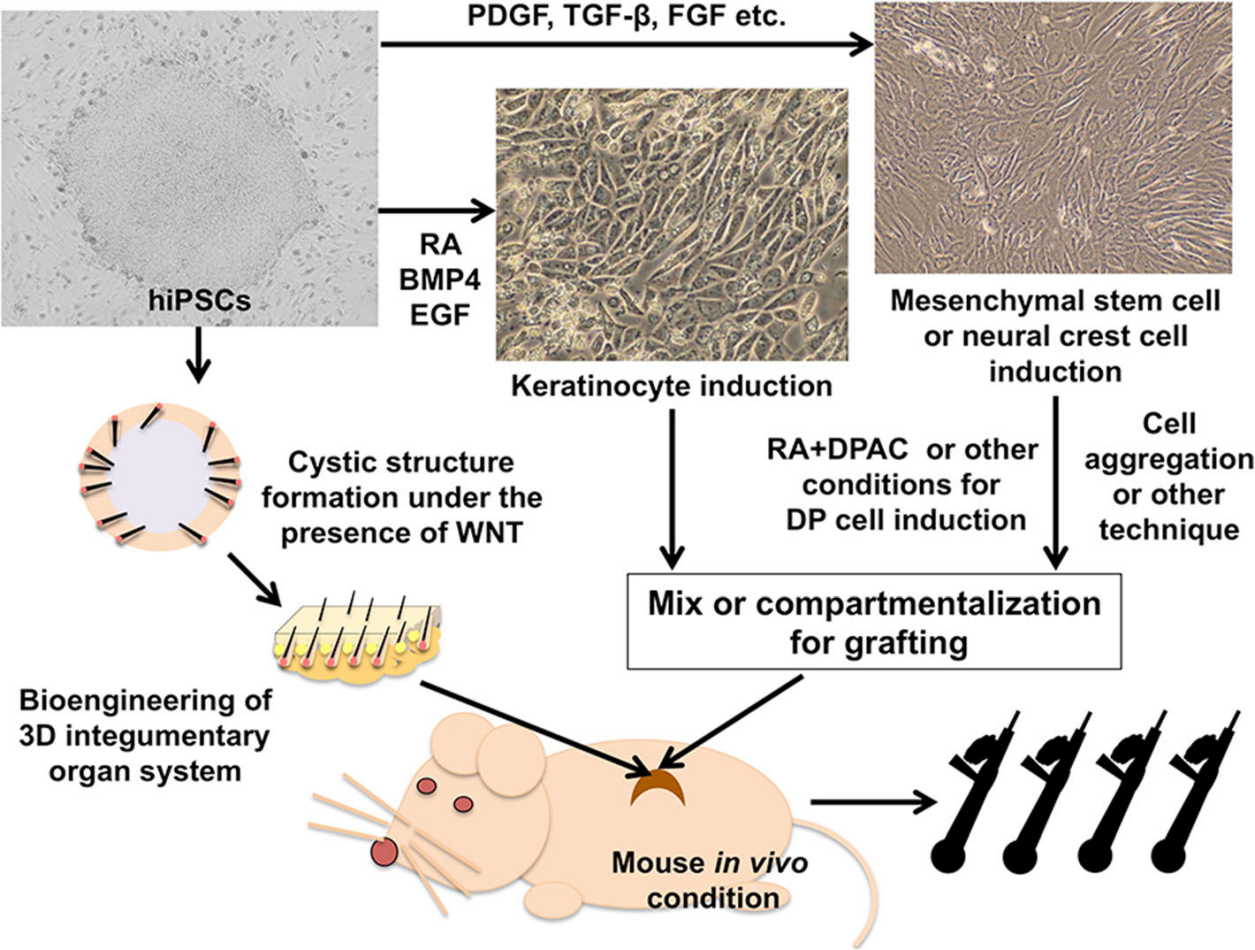
Approaches for HF regeneration using of human induced pluripotent stem cells (hiPSCs). Currently, two respective approaches exist. In the “classic” approach, epithelial and mesenchymal components optimized for trichogenic EMIs are prepared and combined in vivo to form HF structures. Another approach takes advantage of pluripotency of hiPSCs, 3D integumentary organ systems with HFs are microdissected from cystic structures generated by grafting hiPSCs in vivo. HFs can be dissected from 3D integumentary organ systems for downstream applications including hair transplantation

Human embryonic stem cells were differentiated into keratinocytes with retinoic acid (RA) and BMP4 [78, 79] (Fig. 7). Adopting these reagents, fully differentiated KCs were induced from hiPSCs, indicating that KC precursors with fetal KC properties or HFKCs, which are equipped with high receptivity to dermal signals could be generated using hiPSCs [4, 11].

Based on this hypothesis, we induced ectodermal precursor cells (EPCs) which were committed into KC lineage but not terminally differentiated and assessed if they were capable of communicating with DP cells and eventually contribute to HF formation in vivo [11]. In our study, EPCs derived from hiPSCs generated with 4 or 3 Yamanaka factors (*POU5F1*, *SOX2*, *KLF4* +/− *MYC*) expressing keratin 14 and 18 were successfully obtained by the use of RA and BMP4 and KC culture medium. One out of three hiPSC-EPC lines more strongly upregulated HFKC markers than normal human adult KCs (NHKCs), when co-cultured with human DP cells. At the same time, DP cells increased some DP biomarker expression in response to coexisting EPCs. These findings supported that hiPSCs-derived EPCs were capable of eliciting intense EMIs with DP cells. When mixed with trichogenic mice dermal cells and transplanted into nude mice, hiPSC-EPCs, but not NHKCs, partially contributed to regenerated HF structures in the patch assay experiments [11].

Human epithelial HFSCs highly express cell surface markers, such as CD200 and ITGA6 [12, 33]. With some modification to the KC induction protocol followed by selective cell sorting of CD200(+)ITGA6(+) subset, Yang et al. successfully generated epithelial HFSCs [12]. When co-grated with mouse neonatal dermal cells in the chamber assay, generated cells were capable of regenerating HF structures and repopulating into all HF lineages. Moreover, hiPSC-derived HFKCs were shown to differentiate into sebocytes in vitro and reconstituted interfollicur epidermis, indicating their multipotency [12]. These observations indicate that hiPSCs provide materials for the generation of HFSCs with high receptivity to trichogenic dermal signals, which facilitate intense EMIs sufficient for HF bioengineering.

### Generation of DP cells or equivalents from hiPSCs

DP is the vital mesenchymal component for HF morphogenesis and regeneration. However, as mentioned above, currently available approach would not allow preparation of fully functional DP cells for human HF bioengineering urging us to develop a protocol for generating DP or equivalent cells with trichogenicity in vivo [14].

Generation of mesenchymal cells (MCs) with some plasticity from iPSCs has been reported [80, 81]. Thus, we first attempted to establish a methodology to induce MCs with SC properties [14] (Fig. 7). Use of mesenchymal stem cell medium containing PDGF, TGF-β, and FGF enabled the induction of hiPSCs into MCs. LNGFR(+)THY-1(+) subset were subsequently isolated form the induced cell population, which were shown to be highly proliferative and with the capacity to differentiate into osteoblast, adipocyte, and chondrocyte, supporting their mesenchymal SC-like properties [82]. When exposed to RA followed by DPAC in vitro, LNGFR(+)THY-1(+) MCs upregulated some DP biomarkers such as *ALPL*, *WIF1*, *HEY1*, *WNT5A*, *GUCY1A3*, and *LRP4* and were able to bidirectionally communicate with human KCs in co-culture to increase DP and HFKC markers in themselves and KCs respectively (Fig. 7). Importantly, when co-transplanted with human KCs, RA-DPAC-treated LNGFR(+)THY-1(+) MCs gave rise to HF-like structures [14]. Regenerated HF-like structures recapitulated some HF characteristics, including HF-specific marker expression, but were morphologically incomplete and infrequently regenerated. These findings clearly demonstrated that further investigation is necessary to fully establish the methodology to generate DP cells from hiPSCs.

Possible strategies to accomplish this goal include the modification in differentiation protocol including further amelioration of DPAC. Considering that DP cells in the craniofacial area originate from the neural crest [83, 84], induction of DP cells via neural crest cell lineage would be an alternative approach [15]. Taking advantage of this approach, successful HF regeneration using iPS cell-derived epithelial and mesenchymal component has been presented (by Prof. OhSang Kwon, Department of Dermatology, Seoul National University at the World Congress of Hair Research 2017, Kyoto, Japan). The work should move the field forward, when the detailed information is officially published.

### Regeneration of HFs via bioengineered 3D integumentary organ

A unique approach taking full advantage of hiPSCs was recently reported (Fig. 7) [13]. In the study by Takagi and colleagues, 3D integumentary organ system (IOS) was efficiently generated from murine gingiva-derived iPSCs by a transplantation method in which more than 30 embryoid bodies (EBs) were embedded in collagen gel and transplanted into subrenal capsule of severe combined immunodeficient mice [13]. This method allowed formation of cystic areas in transplants at higher ratio than those generated from single iPSC or EB transplants. Intriguingly, 3D IOS, including the skin, HFs, dermis, sebaceous glands, and subcutaneous tissue, were formed at high frequency in explants by CDB transplantation method. In addition, Wnt10b-treatment increased the efficiency of mature HF formation in explants. 3D IOS can be transplanted onto the back of nude mice and regenerated full skin structures containing HFs with normal hair subtype ratio, spacing, and hair cycles. To what extent this new technology is applicable to human iPSCs needs to be further examined. However, this novel approach may enhance opportunity for successful reconstruction of complicated structures from hiPSCs, represented by HFs.

### Future direction of HF bioengineering from hiPSCs

All currently available approaches using iPSCs mentioned above requires in vivo environment to form HF structures [11–15]. Even if HFs of acceptable morphology were successfully made, direct transplantation of regenerated structures into hair loss area of patients is still technically challenging. Reproduction of complete HF structures seems to be straightforward to treat hair loss; however, HF structures are damaged/miniaturized but not lost in the majority of hair loss disorders [85]. Thus, preparation of cell population which supports HF enlargement via cell autonomous or non-autonomous mechanisms, e.g., incorporation into DP or secreting growth factors or activating ligands for signaling pathways crucial for HF neogenesis, could be more practical and cost-friendly approaches which are achievable without in vivo environment [4]. As the width of the hair shafts correlates with the size of DP [86], induction of DS cells with DP precursor cell capacity to repopulate to DP or the cells that constitutively secrete hair growth-promoting factors induced from hiPSCs may represent promising future strategy for the treatment of hair loss diseases. Recent observations, such as restoration of damaged HF in a mouse model with humanized scalp by human DP and DS cells, initiation of new anagen by extracellular vesicles derived from mesenchymal SCs activated DP cells in mice, and promotion of human HF growth by ASCs and their secretary factors [87–89] suggested the possibility of similar approaches using hiPSCs.

It should be noted that the need for regeneration of complete human HF structure is still present. For some types of non-autoimmune-mediated permanent hair loss, represented by scaring alopecia secondary to trauma, burn, or irradiation, and extensive male or female pattern hair loss, transplantation of regenerated HFs would be the only treatment option. For clinical application, in vivo HF reconstitution using immunodeficient mice wound not be preferable mainly because of biosafety. Establishment of in vitro construction of functional HF structures by means of 3D molding (Fig. 3d, e) or IOS (Fig. 7) using hiPSCs-derived HF cells (and possibly HFSPCs as well) is required. The size of bioengineered HFs may be enlarged by the cell-based supplementation strategies as described above.

### Possible application of HFSPCs or hiPSCs-derived HF cells in regenerative medicine

The techniques to better prepare or maintain fully functional HFSPCs or hiPSCs-derived HF cells could also be applied to regenerative medicine for other diseases. In addition to multipotent bulge epithelial HFSPCs capable of repopulating HFs, the epidermis, and sebaceous glands [9] [10], DS cells can be more preferential cell source than dermal fibroblast in the preparation of 3D skin equivalent [90]. Therefore, HFSPCs or hiPSCs-derived HF cells may provide better materials for whole skin regeneration. DP and DS cells were shown to differentiate into hematopoietic, adipogenic, and osteogenic lineages [91, 92]. DP and DS cells supported embryonic stem cells and iPSC maintenance and hematopoiesis in vitro [93]. Furthermore, DS cells exhibited immunosuppressive role to improve islet allograft survival in the mouse model of type Ι diabetes [94]. These observations suggest that HFSPCs or hiPSCs-derived HF cells may provide favorable materials in regenerative medicine not limited to HF bioengineering.

## Conclusion

The advances have been made in the identification of stem/progenitor cell subsets within HFs. Various techniques to enhance EMI and regenerate HFs adopting these subsets have been developed. However, most investigations were conducted in mice. We are aware that mice and human HF cells share fundamental biological properties but they are distinct [95]. Major morphological and physiological characteristics, such as size and hair cycle, are also different between mouse and human HFs [4, 14]. Seemingly, theoretical basis for human HF regeneration have already been developed, yet a pile of problems still remain unsolved before human HF bioengineering becomes truly possible. The wise use of hiPSCs may enhance opportunity to overcome major technical hurdles and enable better understanding of HF biology, drug discovery, and, ultimately, replacement therapy for intractable hair disorders, which can further be applied to treat other tackling diseases.
